# Increased Rac1 activity and Pak1 overexpression are associated with lymphovascular invasion and lymph node metastasis of upper urinary tract cancer

**DOI:** 10.1186/1471-2407-10-164

**Published:** 2010-04-28

**Authors:** Takao Kamai, Hiromichi Shirataki, Kimihiro Nakanishi, Nobutaka Furuya, Tsunehito Kambara, Hideyuki Abe, Tetsunari Oyama, Ken-Ichiro Yoshida

**Affiliations:** 1 Department of Urology, Dokkyo Medical University, Mibu, Tochigi, Japan; 2 Department of Molecular and Cell Biology, Dokkyo Medical University, Mibu, Tochigi, Japan; 3 Department of Anatomic and Diagnostic Pathology, Dokkyo Medical University, Mibu, Tochigi, Japan

## Abstract

**Background:**

Lymphovascular invasion (LVI) and lymph node metastasis are conventional pathological factors associated with an unfavorable prognosis of urothelial carcinoma of the upper urinary tract (UC-UUT), but little is known about the molecular mechanisms underlying LVI and nodal metastasis in this disease. Rac1 small GTPase (Rac1) is essential for tumor metastasis. Activated GTP-bound Rac1 (Rac1 activity) plays a key role in activating downstream effectors known as Pak (21-activated kinase), which are key regulators of cytoskeletal remolding, cell motility, and cell proliferation, and thus have a role in both carcinogenesis and tumor invasion.

**Methods:**

We analyzed Rac1 activity and Pak1 protein expression in matched sets of tumor tissue, non-tumor tissue, and metastatic lymph node tissue obtained from the surgical specimens of 108 Japanese patients with UC-UUT.

**Results:**

Rac1 activity and Pak1 protein levels were higher in tumor tissue and metastatic lymph node tissue than in non-tumor tissue (both *P *< 0.0001). A high level of Rac1 activity and Pak1 protein expression in the primary tumor was related to poor differentiation (*P *< 0.05), muscle invasion (*P *< 0.01), LVI (*P *< 0.0001), and lymph node metastasis (*P *< 0.0001). Kaplan-Meier survival analysis showed that an increase of Rac1 activity and Pak1 protein was associated with a shorter disease-free survival time (*P *< 0.01) and shorter overall survival (*P *< 0.001). Cox proportional hazards analysis revealed that high Rac1 activity, Pak1 protein expression and LVI were independent prognostic factors for shorter overall and disease-free survival times (*P *< 0.01) on univariate analysis, although only Pak1 and LVI had an influence (*P *< 0.05) according to multivariate analysis.

**Conclusions:**

These findings suggest that Rac1 activity and Pak1 are involved in LVI and lymph node metastasis of UC-UUT, and may be prognostic markers for this disease.

## Background

Urothelial carcinoma of the upper urinary tract (UC-UUT) is relatively uncommon, accounting for <10% of all urothelial malignancies, but its incidence is increasing [1]. Many patients who undergo curative resection develop systemic metastases within a few years, so the prognosis of this cancer is poor [2], presumably due to occult micrometastasis at the time of surgery because of the thin walls and rich lymphatic drainage of the ureter.

Metastasis involves the spread of tumor cells from the primary tumor to a distant site [3], and is the major cause of human cancer death. Various pathological studies have shown that poorly differentiated cancer, muscle invasion, lymph node metastasis, and lymphovascular invasion (LVI) are associated with recurrence and are unfavorable prognostic factors for UC-UUT [2,4,5]. Thus, LVI and lymph node metastasis are used to predict the prognosis. Despite their clinical importance, little is known about the molecular mechanisms of LVI and lymph node metastasis, making it important to examine the factors playing a role in LVI and lymph node metastasis of UC-UUT.

Members of the Rho small GTPases family, prototype RhoA, Rac1, and Cdc42, are involved in the regulation of a variety of cellular processes, such as organization of the microfilament network, cell-cell contact, and malignant transformation, and also perform essential and specialized functions during organization of the actin cytoskeleton [6]. RhoA regulates the formation of stress fibers and focal adhesions in cells, while Rac1 regulates the formation of lamellipodia and membrane ruffling, and Cdc42 regulates the formation of filopodia [6,7]. In addition, a number of investigations have established a significant role of GTPases from the Rho family in several human tumors, including UC-UUT [7,8]. Rac1 is ubiquitously expressed and exists in two conformational states. In response to extracellular signals, interconversion of these two states occurs via guanine nucleotide exchange factors (GEFs), which convert the inactive GDP-bound form of Rac1 to its active GTP-bound form, while GTPase-activating proteins (GAPs) inactivate proteins (GAPs) inactivate Rac1. After activation, Rac1 interacts with various specific effectors to coordinate the activation of a multitude of signaling cascades that influence diverse physiological outcomes. The Pak (p21-activated kinase) serine/threonine kinases have recently been found to be key regulators of cytoskeletal remolding, cell motility, and cell proliferation, with a role in both carcinogenesis and cellular invasion [9]. It has been reported that Pak1, the best characterized member of this family, shows increased expression and activity in human cancers [9-11]. Multiple signalling pathways converge to promote activation of Pak1 through both small GTPases and several of the tyrosine kinases. In turn, activated Pak1 regulates diverse cellular functions. Pak1 binds to Rac1 in a GTP-dependent manner, after which activated Pak1 regulates cellular functions such as cytoskeletal dynamics, cell adhesion, and transcription [9]. Rac1 signals through Pak1 to activate c-Jun N-terminal kinase (JNK) [9], placing Rac1 between the Ras small GTPases (Ras) and mitogen-activated protein kinase (MAPK) in the signaling cascade from growth factor receptors and v-Src to activation of JNK [12,13]. Gao et al. reported that a low molecular weight inhibitor of Rac GTPase targeting the activation of Rac by GEF was able to reverse the tumor cell phenotype associated with deregulation of Rac [14]. In addition, several low molecular weight inhibitors have been shown to interfere with Pak kinase activity or function [9]. These findings suggest that Rac1 and Pak may be potential molecular targets for the treatment of cancer.

Regarding the expression of Rho family GTPases in human cancers, most previous reports were based on the investigation of protein expression levels by Western blotting and immunohistochemistry. However, only the active GTP-binding form (active GTPase) recognizes target proteins and generates a response. Increased activity of Rac1 and overexpression of Pak1 are associated with the progression of cancer, but most of the evidence has come from cell culture studies. Therefore, the role of active Rac1 GTPase and its downstream effector needs to be studied by using surgically resected samples of human tumors to better assess their contribution to human cancer. Accordingly, we examined the expression of active GTP-bound Rac1 (Rac1 activity) and its downstream effector Pak1 in the primary tumors and metastatic lymph nodes of patients with UC-UUT by Western blotting, and also assessed the relation of these molecules with clinicopathological features. There have been few reports about the simultaneous analysis of Rac1 and Pak1 in human tumor tissues. Such information could be useful for developing individualized treatment strategies and could potentially improve the design and application of adjuvant therapy for UC-UUT.

## Methods

### Patients and tissues

Between 1995 and 2006, surgical specimens of UC-UUT were obtained from 108 consecutive Japanese patients (77 men and 31 women) aged 42 to 89 years (mean age: 71.9 years) with newly diagnosed primary transitional cell carcinoma (TCC) of the renal pelvis and ureter without distant metastasis (cT_any_N_any_M0). These specimens were reviewed in the present study. All patients routinely underwent imaging investigations (CT and/or MRI) before surgery to acquire information for staging. The follow-up time ranged from 5 to 132 months, with a median follow-up period of 41 months. Patients underwent surgery before receiving any other therapy.

During nephroureterectomy, the patients also underwent lymphadenectomy when enlarged lymph nodes were confirmed. In all patients, tumor tissue, non-tumor tissue, and lymph node tissue were acquired from the resected specimens after removing excess stromal tissue. Then the tissues were embedded in OCT tissue compound (Miles, Elkhart, IN, USA) and stored at - 80°C according to the method described previously [15]. The grade and stage of each tumor were classified according to the TNM system [16]. This study was conducted in accordance with the Helsinki Declaration and institutional review board approval in Dokkyo Medical University Hospital was obtained. In addition, each patient signed a consent form approved by the Committee on Human Rights in Research of our institution.

Systemic chemotherapy with methotrexate, vinblastine, Adriamycin, and cisplatin (M-VAC) was given to the 16 patients who had lymph node metastasis at nephroureterectomy, as well as those in whom lymph node metastasis (14 patients) or distant metastasis (5 patients) was detected postoperatively [17].

### Rac1 activation assay and Western blotting

Tumor tissue and normal tissue specimens were carefully dissected free of stromal tissue. To measure Rac1 activation, we performed a Rho-binding domain (RBD) affinity precipitation assay for Rac1-GTP using a specific Rac1 antibody according to the manufacturer's protocol (Cytoskeleton, BK126, Denver, CO) [18-20]. Tissue extracts were obtained from 50 mg of each type of tissue and the protein concentration was determined by Bradford's method (BIO-RAD, Hercules, CA). Then the extracts were equalized with ice cold cell lysis buffer containing 25 mM Tris (pH 7.5), 10 mM MgCl_2_, 0.5 M NaCl, and 1% Triton X-100 to obtain identical protein concentrations. Next, equivalent amounts of protein were added to 15 μl of Pak (p21-activated kinase)-RBD beads and incubated at 4°C on a rotor for 1 hr. Then the Pak-RBD beads solution was centrifuged at 3,000 *g *for 1 min at 4°C and supernatant was removed. After washing the pellet three times, the bound proteins were analyzed by Western blotting, as described previously [21]. Briefly, 25 μg of GTP-Rac1 bound to Pak-RBD beads was separated by SDS-PAGE (5-20% gradient gel) and electrotransferred to a polyvinylidene difluoride membrane (Sequi-Blot PVDF membranes; BIO-RAD). Rac1 His-tagged protein (100, 50, and 10 ng/μl) was also run on the gel as a standard. If the intensity of the immunoreactive band was outside the range of 10-100 ng/μl, samples were diluted with loading buffer and run on the gel again. Total cell lysate with GDP was employed as a negative control. After membranes were blocked to eliminate nonspecific binding, membrane-bound proteins were probed with an anti-Rac1 monoclonal antibody (Cytoskeleton, BK126). Then the membranes were washed and incubated with a horseradish peroxidase-conjugated secondary antibody. Antibody-bound protein bands were visualized by chemiluminescence, the blotted membrane was subjected to densitometry by scanning with a ChemiDoc XRS-J imaging scanner (BIO-RAD), and the data were analyzed with NIH Image software. The mean value for three experiments was obtained with each tissue specimen.

For measurement of Pak1, 50 μg of cytosolic protein was separated by SDS-PAGE (12.5% gel) and electrotransferred to a polyvinylidene difluoride membrane (Immobilon-P membrane; Millipore, Bedford, MA). After the membrane was blocked, the bound proteins were probed with specific antibodies (Santa Cruz Biotechnology; sc-881, Santa Cruz, CA) and a primary antibody for beta actin (Santa Cruz Biotechnology, Santa Cruz, CA). Hela cells were used as a positive control for Rac1 and Pak1 expression. Next, the membranes were washed and then incubated with horseradish peroxidase-conjugated secondary antibodies. Antibody-bound protein bands were visualized by chemiluminescence, the blotted membrane was scanned for densitometry with a PDI imaging scanner (Agfa Japan, Tokyo), and the data were analyzed with NIH Image software. Expression of active Rac1 and Pak1 was determined relative to that of beta actin in the tumor tissue and corresponding normal tissue specimens, after which relative expression was calculated. For quantification of these proteins, the relative amount of Rac1 or Pak1 in tumor tissue was expressed as a ratio of the optical density of the band obtained from the tumor specimen to that from the corresponding normal tissue (which was set at 1.0) by densitometric analysis as described previously [21-23]. The mean values for tumor tissue and non-tumor tissue were calculated from three experiments [21,22].

### Immunohistochemistry

To support the data obtained by Western blotting, immunohistochemistry was performed with the same antibodies utilized for Western blotting on 3 tumors from patients with lymph node metastases (pN+) and 5 tumors from patients without nodal metastasis (pN-), as described previously [20-22].

### Statistical analysis

Western blotting data were analyzed by the Mann-Whitney *U *test to compare two groups [21,22], or by the Kruskal-Wallis test for comparisons among three or more groups. Spearman's rank correlation coefficient analysis was employed to determine the relation between Rac1 activity and Pak1 expression. Rac1 activity and Pak1 expression, as well as tumor grade, pT stage, lymph node metastasis, and LVI, were assessed for their relation to disease-free survival and overall survival by univariate and multivariate analyses with the Cox proportional hazards model. The Kaplan-Meier method was used to estimate survival, and differences of survival were assessed by the log-rank test. Probability values of less than 0.05 were considered significant. Data were analyzed with commercially available software.

## Results

### Association of Rac1 activity and Pak1 expression with tumor characteristics

**Figure 1:**
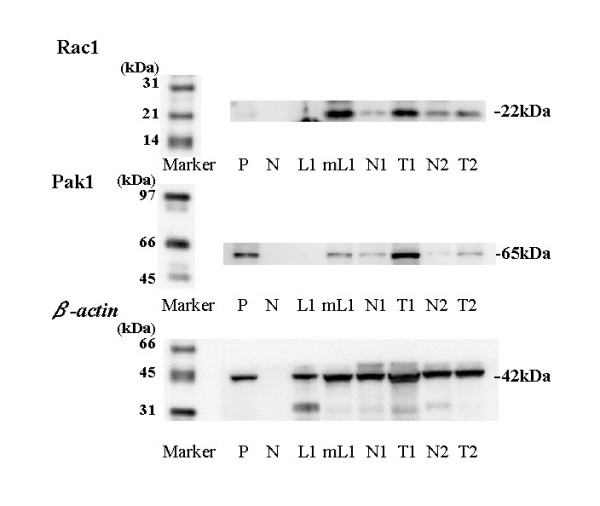
**Expression of GTP-binding (active form) Rac1 (22 kDa), Pak1 (65 kDa) and beta actin (42 kDa) proteins using Western blotting**. M; marker, P; positive control using Hela cells, N; negative control, T1,2; tumor tissue, N1,2; non-tumor tissue. LN1; non-tumor lymph node, mLN1; metastatic lymph node, Each number corresponds to a case number.

**Figure 2:**
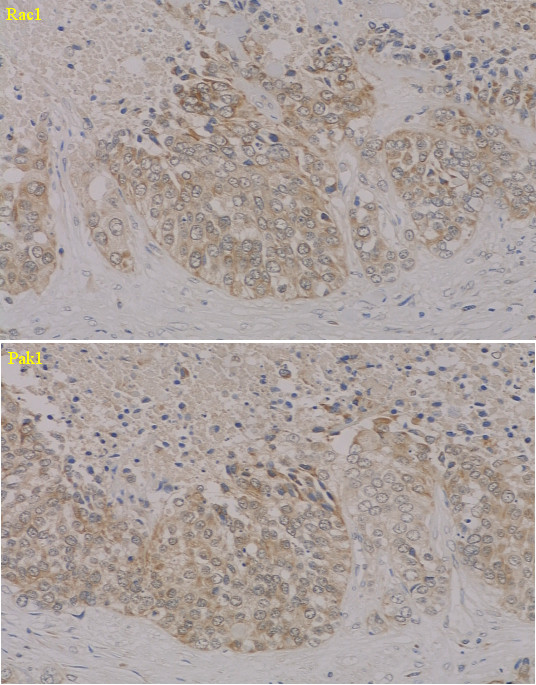
**Immunohistochemistry for Rac1 (upper panel) and Pak1 (lower panel) proteins in Grade 3 carcinoma**. Cytosolic compartments shows intensely brown staining in most of the cancer cells, displaying high Rac1 and Pak1 protein levels, but the nuclei of the cancer cells shows very weak staining.

**Figure 3:**
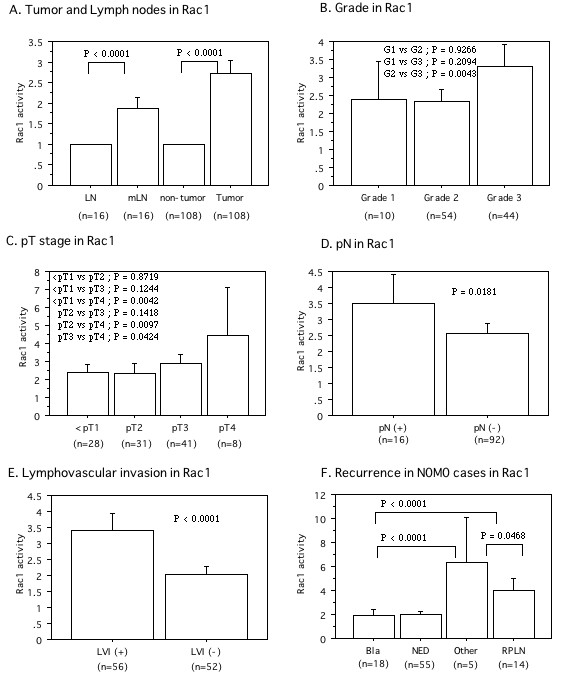
**The relative expression levels of GTP-binding active Rac1 protein in tumor to those in corresponding non-tumor portion, which was set to 1.0**. A; Expression in tumor, non-tumor, and lymph node tissues with metastasis (mLNs) and without (LN). B; Expression in Grade. C; Expression in pT stage. D; Expression in lymph node metastasis. pN(-) is pN0. pN(+) is pN1-3. E; Expression in lymphovascular invasion (LVI). F; Expression in first recurrence site. There were no difference between bladder and none of recurrence. The data show the 95% confidential interval.

Rac1 and Pak1 proteins were detected in tumor tissues, non-tumor tissues, metastatic lymph nodes, and normal lymph nodes (Figures. 1, 2). The level of active (GTP-bound) Rac1 was significantly increased in tumor tissues (mean ± S.D. = 2.72 ± 1.72) and metastatic lymph nodes (1.86 ± 0.34) compared with the level in non-cancerous tissues, which was set at 1.0 [21-23], (P < 0.0001, Figure. 3A). An increase of Rac1 activity in primary tumors was associated with poorly differentiated cancer (grade 1; 2.38 ± 1.38, grade 2; 2.33 ± 1.31, grade 3; 3.30 ± 2.08, P = 0.0471, Figure. 3B), local invasion (<pT1; 2.38 ± 1.35, pT2; 2.32 ± 1.31, pT3; 2.91 ± 1.69, pT4; 4.46 ± 3.14, P = 0.1417, Figure. 3C), lymph node metastasis (pN0; 2.55 ± 1.62, pN1-3; 3.52 ± 1.96, P < 0.05, Figure. 3D), and LVI (LVI(-); 2.04 ± 0.97, LVI(+); 3.40 ± 2.02, P < 0.0001, Figure. 3E).

**Figure 4:**
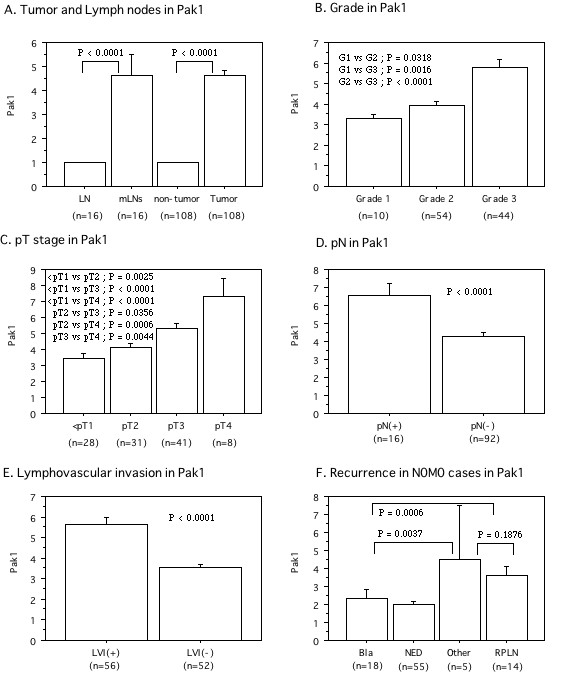
**The relative expression levels of Pak1 protein in tumor to those in corresponding non-tumor portion, which was set to 1.0**. A; Expression in tumor, non-tumor, and lymph node tissues with metastasis (mLNs) and without (LN). B; Expression in Grade. C; Expression in pT stage. D; Expression in lymph node metastasis. pN(-) is pN0. pN(+) is pN1-3. E; Expression in lymphovascular invasion (LVI). F; Expression in first recurrence site. There were no difference between bladder and none of recurrence. The data show the 95% confidential interval.

The level of Pak1 protein was significantly higher in tumor tissues (mean ± S.D. = 2.68 ± 1.26) and metastatic lymph nodes (2.77 ± 1.60) than the level in non-cancerous tissues, which was also set at 1.0 [21-23], (*P *< 0.0001, Figure. 4A). Higher expression of Pak1 protein in the primary tumor was associated with poorly differentiated cancer (grade 1; 1.67 ± 0.34, grade 2; 2.38 ± 0.96, grade 3; 3.29 ± 1.45, *P *< 0.0001, Figure. 4B), local invasion (<pT1; 1.89 ± 0.60, pT2; 2.52 ± 0.97, pT3; 3.09 ± 1.12, pT4; 4.56 ± 2.10, *P *< 0.0001, Figure. 4C), lymph node metastasis (pN0; 2.43 ± 1.09, pN1-3; 3.84 ± 1.35, *P *< 0.0001, Figure. 4D), and LVI (LVI(-); 2.03 ± 0.68, LVI(+); 3.34 ± 1.36, *P *< 0.0001, Figure. 4E).

**Figure 5:**
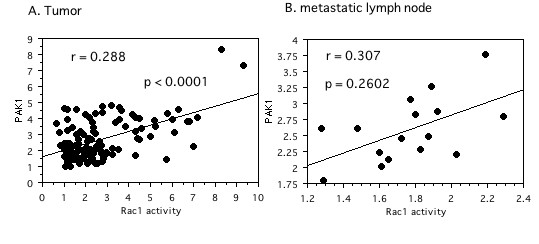
**Spearman rank correlation coefficient relationship between expression levels of Rac1 activity and Pak1**. X axis is an independent variable. Y axis is a dependent variable. A; Tumor tissues. B; Metastatic lymph node tissues.

We investigated the correlation between Rac1 activity and Pak1 expression in tumor tissues. When Rac1 was used as an independent variable and Pak1 as a dependent variable, a positive correlation between them was observed (r^2 ^= 0.288, *P *< 0.0001, Figure. 5A). However, no such correlation was observed in specimens of metastatic lymph nodes (r^2 ^= 0.307, *P *= 0.2602, Figure. 5B).

### Prognostic influence of Rac1 activity and Pak1 expression

The mean level of Rac1 activity was 2.72 (± 1.72). Patients were divided into two groups at this value, i.e., a high activity group (46 patients) and a low activity group (62 patients), according to the method described previously [21-23]. Similarly, the mean level of Pak1 protein expression in the tumor tissues was 2.68 (± 1.26), so a high expression group (49 patients) and a low expression group (59 patients) were separated by using this as the cut-off value.

**Figure 6:**
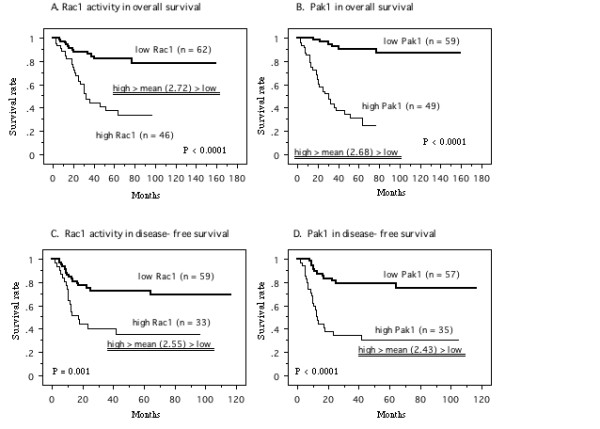
**Survival curve in the patients based on the mean values of Rac1 activity and Pak1 in primary tumor tissues, the cases were divided into two groups at this levels - high and low expression**. A; Overall survival curve based on Rac1 activity in all patients. B; Overall survival curve based on Pak1 in all patients. C; Disease-free survival curve based on Rac1 activity in N0 M0 patients at nephroureterectomy. D; Disease-free survival curve based on Pak1 in N0 M0 patients at nephroureterectomy. *P *value was analyzed by log-rank test.

Kaplan-Meier plots of survival for patients with low versus high levels of Rac1 activity showed that increased Rac1 activity was associated with a shorter overall survival time (*P *< 0.0001, Figure. 6A). High expression of Pak1 protein was also correlated with shorter overall survival (*P *< 0.0001, Figure. 6B). Univariate analysis of overall survival with the Cox proportional hazards model revealed that tumor grade, pT stage, lymph node metastasis, LVI, active Rac1, and Pak1 were all significant variables (Table 1). However, multivariate analysis revealed that only LVI and Pak1 had an independent influence on overall survival.

**Table 1:** Cox regression analysis for various potential prognostic factors in survival

			Overall survival in all patients					Disease-free survival in N0 M0 patients at nephroureterectomy			
		
Variable	Unfavorable/favorable characteristics	No. of Patients	Analysis	Relative risk	95% confidential interval	P value	No. of Patients	Analysis	Relative risk	95% confidential interval	P value
			Univariate (U)	2.626	1.486 - 4.641	0.0009		Univariate (U)	1.947	1.117-3.392	0.0187
**Grade**	3/2/1	44/54/10					32/51/9				
			Multivariate (M)	1.008	0.488 - 2.083	0.6126		Multivariate (M)	1.047	0.427 - 1.678	0.6332

			U	3.138	2.065 - 4.768	< 0.0001		U	1.905	1.308 - 2.774	0.0008
**pT**	4/3/2/1 >	8/41/31/28					3/37/26/26				
			M	1.499	0.837 - 2.526	0.1454		M	1.291	0.479 - 1/302	0.3551

			U	3.049	2.005 - 4.636	< 0.0001					
**pN**	(+)/(-)	16/92									
			M	1.48	0.894 - 2.448	0.1271					

			U	11.024	5.550 - 21.896	< 0.0001		U	10.298	4.737 - 22.388	< 0.0001
**LVI**	(+)/(-)	56/52					40/52				
			M	6.923	2432 - 19.707	< 0.0001		M	9.954	4.093 - 24.204	< 0.0001

			U	4.363	2.188 - 8.702	< 0.0001		U	2.917	1.494 - 5.694	0.0017
**Rac1**	high/low	46/62					33/59				
			M	1.87	0.863 - 4.094	0.1223		M	1.323	0.627 - 2.791	0.4621

			U	12.633	4.869 - 32.776	< 0.0001		U	4.814	2.384 - 9.721	< 0.0001
**Pak1**	high/low	49/59					35/57				
			M	3.635	1.230 - 10.741	0.0196		M	3.526	1.564 - 7.946	0.0024

With regard to disease-free survival of the patients who were N0 M0 at the time of nephroureterectomy (92 patients), the mean level of Rac1 activity and Pak1 expression in tumor tissue was 2.55 ± 1.62 and 2.43 ± 1.09, respectively. Comparison of Kaplan-Meier plots for the N0 M0 patients with low Rac1 activity (59 patients) versus high Rac1 activity (33 patients) showed that high Rac1 activity was associated with a shorter disease-free survival time (*P *= 0.001, Figure. 6C). Similarly, higher expression of Pak1 was a significant unfavorable factor for disease-free survival (*P *< 0.0001, Figure. 6D). Although tumor grade, pT stage, LVI, active Rac1, and Pak1 were all significant factors by Cox univariate analysis, only LVI and Pak1 were independent variables according to multivariate analysis (Table 1).

**Figure 7:**
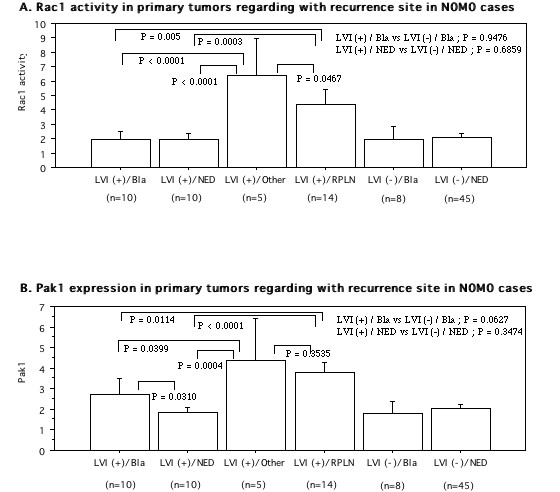
**Rac1 activity (A) and protein levels of Pak1 (B) regarding with postoperative first recurrence site among N0 M0 cases (92 patients)**. The data show the 95% confidential interval.

With regard to the site of first postoperative recurrence among the 92 N0 M0 patients, the Rac1 activity and Pak1 protein levels in the primary tumor tissues of patients with retroperitoneal lymph node metastasis (PRLN) (n = 14; 3.99 ± 1.82, 3.61 ± 0.94, respectively) and other organ metastases (3 of lung, one of liver, and one of bone; 6.35 ± 2.35 and 4.49 ± 1.90, respectively) were significantly higher than the levels in patients with bladder recurrence (n = 18; 1.95 ± 0.89, *P *< 0.0001, 2.32 ± 1.02, *P *< 0.01, respectively, Figures. 3F, 4F). Furthermore, we divided this group into two subgroups to study the relationship between LVI and Rac1 activity or Pak1 expression (Figure. 7). When the PRLN and distant organs were the first site of recurrence, the primary tumors had LVI, while primary tumors without LVI showed no PRLN recurrence or distant metastasis. Among the LVI(+) group, both Rac1 activity and Pak1 expression in the primary tumors were higher in patients with RPLN and distant metastases than in those with bladder recurrence. The patients with bladder recurrence had higher tumor levels of Pak1 expression than those with no evidence of disease (NED), but Rac1 activity did not vary. On the other hand, in the LVI(-) group, both Rac1 activity and Pak1 expression were not different between the primary tumors of patients with bladder recurrence and those with NED. Interestingly, although Rac1 activity showed no difference between LVI(+) and LVI(-) tumors in patients with bladder recurrence, Pak1 expression was higher in the former tumors.

M-VAC therapy was performed for 16 patients with lymph node metastasis at the time of nephroureterectomy, 14 patients with postoperative lymph node involvement, and 5 patients with postoperative metastasis to other organs. All of these 35 patients eventually died of progressive cancer. Their primary tumors were all LVI(+) on pathological examination, as well as showing increased Rac1 activity and high Pak1 expression (data not shown).

## Discussion

Rac1 and Pak1 have recently been shown to be key regulators of cancer cell signaling networks, and there are several lines of evidence linking Rac1 and Pak1 to the acquisition of migratory, invasive, and metastatic phenotypes [7,9]. In order to take into account possible inter-individual variations of Rac1 activity and Pak1 protein expression in UC-UUT, we performed a comparison among paired samples of tumor tissue, metastatic lymph node tissue, and non-tumor tissue from the same patient. The present study showed that Rac1 activity and Pak1 expression were significantly increased in primary tumors and metastatic lymph nodes compared with non-tumor tissues. Also, an increase of Rac1 activity and Pak1 expression in the primary tumor was correlated with poorly differentiated cancer, local invasion, lymph node metastasis, LVI, and an unfavorable prognosis. To our knowledge, this is the first report about Rac1 and Pak1 in UC-UUT. Our data suggested that Rac1 and its downstream effector Pak1 may be involved in the progression of this cancer.

As well as our findings in patients with UC-UUT, overexpression of Rac1 and Pak1 has been reported in several other human cancers [7,9]. Moreover, an increase of Rac1 and Pak1 activity or overexpression have been observed in breast cancer tissues and metastatic lymph nodes [24,25]. In the present study, increased Rac1 activity and higher Pak1 expression in the primary tumors was related to muscle invasion and lymph node metastasis. Therefore, it is likely that Rac1 and Pak1 have a role in determining the local invasive and metastatic potential of various human cancers.

Regarding the site of initial postoperative recurrence in patients who were pT_any_pN0 M0 at the time of nephroureterectomy, patients with postoperative lymph node recurrence had a worse prognosis than those with bladder recurrence, probably because many bladder cancers were detected at the superficial stage by active surveillance. In the present study, LVI, Pak1 activity, Rac1, pT stage, and tumor grade were related to postoperative recurrence according to univariate analysis, with both LVI and Pak1 still being significant determinants according to multivariate analysis. As shown in Figure 7, Rac1 activity and Pak1 expression were higher in the primary tumors of patients with postoperative lymph node metastasis than in those of patients with bladder recurrence from the LVI(+) group, but not the LVI(-) group. Pak1 expression was higher in the tumors of patients with bladder recurrence than in recurrence-free patients from the LVI(+) group, but Rac1 did not differ between them. Moreover, all patients with lymph node involvement at nephroureterectomy had LVI(+) tumors on pathological examination. Therefore, LVI might be an important step along the road to lymph node metastasis. Primary tumors with LVI(+) and lymph node metastasis showed an increase of Rac1 activity and Pak1 expression, while metastatic lymph node tissues showed higher Rac1 activity and Pak1 expression than normal lymph nodes, indicating that Rac1 and Pak1 are involved in tumor metastasis. However, it is unclear whether Rac1 and Pak1 play a similar role in lymph node metastasis and bladder recurrence. It is well known that urothelial cancer often behaves like a field change disease, with multiple occurrences and recurrences due to implantation and migration, making it difficult to determine whether a recurrent tumor has been caused by tumor cell implantation, migration, or multifocal carcinogenesis [26]. A recent molecular study revealed that UC-UUT might be less genetically stable than bladder tumors [27]. Therefore, an increase of Rac1 activity and upregulation of Pak1 expression might play a role in lymph node metastasis of UC-UTT after nephroureterectomy, rather than contributing to bladder recurrence. The differences of molecular mechanisms between LVI and lymph node metastasis or bladder recurrence need to be investigated further. On the other hand, a previous study of bladder cancer showed that high Pak1 expression was associated with a higher risk of recurrence, even in patients with low grade/stage tumors [28]. Taken together, therefore, it is likely that Pak1 and Rac1 both play an important role in the invasion, metastasis, and recurrence of urothelial cancer.

Although Pak1 is well known as a downstream effector of Rac1, there have been few simultaneous analyses of Rac1 and Pak1 expression in human tumor tissues. Our study showed a positive correlation between Rac1 activity and Pak1 expression in tumor tissue, while no such relation was observed in metastatic lymph node tissue. In contrast to investigation of Rac1 activity, we only measured Pak1 protein expression, but we could still determine the approximate relation between the two molecules. As shown in Figure 5, there was a positive correlation in tumor tissues, but the absolute correlation was fairly weak and no correlation was found in metastatic lymph nodes. Pak1 is the best-characterized downstream effector of Rac1, but it is also an important convergence point for many signaling pathways (including small GTPases and several tyrosine kinases) that are often activated in cancer cells [9-11]. Therefore, several oncogenic pathways may act through Pak1 to promote cancer progression, so that Pak1 protein expression had a greater impact on overall and recurrence-free survival than Rac1 activity in the present study. We did not assess Pak1 activity in this study, so its activity in tumor tissues needs to be elucidated in the future.

Cell migration is central to metastasis by malignant tumors [3]. Members of the Rho small GTPases family regulate formation of stress fibers, focal adhesions, and cell migration through reorganization of the actin cytoskeleton [6]. Several lines of evidence have directly linked Rac1 and Pak1 to acquisition of a migratory, invasive, and metastatic phenotype and to a variety of processes that occur in tumors, including cell transformation, survival, invasion, metastasis, and angiogenesis [7,9]. Our findings suggested that Rac1 and Pak1 were associated with LVI and RPLN, as well as distant metastasis of UC-UUT.

The renal pelvis and ureter have thin walls and a rich lymphatic drainage [1], so many patients present with local invasion and/or lymph node metastasis, while delay in making the initial diagnosis is correlated with a higher stage at presentation. Preoperative staging by CT/MRI was very useful for detecting patients with local invasion and/or lymph node metastasis in the present study, but the prognosis of such patients was poor (Table 1). Although systemic M-VAC therapy reduced the tumor burden of our patients with urothelial cancer, the prognosis was worse than we expected [17]. In the present study, M-VAC therapy was performed as adjuvant chemotherapy for the 35 patients who showed lymph node or distant metastasis at surgery or during postoperative surveillance, but all of these patients died of their cancer. These 35 tumors were all LVI(+) and showed increased Rac1 activity and high Pak1 expression. Furthermore, an increase of Rac1 activity and a higher Pak1 expression were associated with a shorter overall survival time in all patients and shorter postoperative disease-free survival in pT_any_pN0 M0 patients, indicating that Rac1 activity and Pak1 expression may be useful prognostic indicators for UC-UTT. These findings suggests that patients with LVI(+) tumors that have higher Rac1 activity and Pak1 expression are at more risk of developing postoperative RPLN or distant metastases in comparison to patients without these markers. Therefore, we need to assess the potential of chemotherapy for patients who have pT_any_pN0 M0 tumors that are LVI(+) with increased Rac1 activity and Pak1 expression to prevent RPLN recurrence or distant metastasis by performing a randomized study in the future. In premenopausal breast cancer patients, Pak1 overexpression has been closely linked with tamoxifen resistance of tumors [29]. On the other hand, several low molecular weight inhibitors have been shown to interfere with Pak1 kinase activity or function [9]. Therefore, the Rac1-Pak1 pathway might be a potential therapeutic target for the prevention of tumor invasion and metastasis by inhibition of this signaling pathway. Accordingly, we should examine the effects of various inhibitors using cell lines or tumor tissue samples. Furthermore, as the Rac family has three isoforms and the Pak family has six isoforms, each isoform may play a different role. Therefore, it is necessary to investigate each of these isoforms in human cancers, and the information thus obtained may shed new light on treatment strategies for UC-UTT and other tumors.

## Conclusion

In the present study, increased Rac1 activity and increased expression of Pak1 (a major downstream effector) were associated with poorly differentiated tumors, local invasion, LVI, lymph node metastasis, and an unfavorable prognosis. Our findings suggest that the Rac1-Pak1 pathway may be related to the progression of UC-UUT and that these molecules may be indicators for this disease.

## Competing interests

The authors declare that they have no competing interests.

## Authors' contributions

T.K.* and H.S. initiated the study, participated in its design and coordination, carried out the study, performed the statistical analysis, and drafted the manuscript. K.N., N.F., T.K., H.A. and T.O. carried out the study. K-I.Y. participated in the design of the study and helped to draft the manuscript. All authors read and approved the final manuscript.

## Pre-publication history

The pre-publication history for this paper can be accessed here:

http://www.biomedcentral.com/1471-2407/10/164/prepub
